# Effects of OEF/OIF-Related Physical and Emotional Co-Morbidities on Associative Learning: Concurrent Delay and Trace Eyeblink Classical Conditioning

**DOI:** 10.3390/ijerph110303046

**Published:** 2014-03-12

**Authors:** Regina E. McGlinchey, Catherine B. Fortier, Jonathan R. Venne, Arkadiy L. Maksimovskiy, William P. Milberg

**Affiliations:** 1 Geriatric Research Education and Clinical Center (GRECC) and the Translational Research Center for TBI and Stress-related Disorders (TRACTS), Boston, MA 02130, USA; E-Mails: Catherine_Fortier@hms.harvard.edu (C.B.F.); jon.venne@gmail.com (J.R.V.); arkadiym@bu.edu (A.L.M.); William_Milberg@hms.harvard.edu (W.P.M.); 2 Department of Psychiatry, Harvard Medical School, Boston, MA 02115, USA; 3 Department of Behavioral Neuroscience, Boston University School of Medicine, Boston, MA 02118, USA

**Keywords:** posttraumatic stress disorder, mild traumatic brain injury, alcohol

## Abstract

This study examined the performance of veterans and active duty personnel who served in Operation Enduring Freedom and/or Operation Iraqi Freedom (OEF/OIF) on a basic associative learning task. Eighty-eight individuals participated in this study. All received a comprehensive clinical evaluation to determine the presence and severity of posttraumatic stress disorder (PTSD) and traumatic brain injury (TBI). The eyeblink conditioning task was composed of randomly intermixed delay and trace conditioned stimulus (CS) and unconditioned stimulus (US) pairs (acquisition) followed by a series of CS only trials (extinction). Results revealed that those with a clinical diagnosis of PTSD or a diagnosis of PTSD with comorbid mTBI acquired delay and trace conditioned responses (CRs) to levels and at rates similar to a deployed control group, thus suggesting intact basic associative learning. Differential extinction impairment was observed in the two clinical groups. Acquisition of CRs for both delay and trace conditioning, as well as extinction of trace CRs, was associated with alcoholic behavior across all participants. These findings help characterize the learning and memory function of individuals with PTSD and mTBI from OEF/OIF and raise the alarming possibility that the use of alcohol in this group may lead to more significant cognitive dysfunction.

## 1. Introduction

Emerging evidence suggests that there is a significant overlap in the clinical symptoms associated with posttraumatic stress disorder (PTSD) and mild traumatic brain injury (mTBI), conditions that co-occur with great frequency in veterans of Operation Enduring Freedom and Operation Iraqi Freedom (OEF/OIF). Neuropsychological measures have had mixed success in identifying the unique contributions of these two disorders to the overall picture of deficits in cognitive and emotional function. The focus of the present study was to examine elemental memory function in OEF/OIF veterans using classical or Pavlovian conditioning, which is an associative learning paradigm that, relative to more complex learning and memory tasks, is much less susceptible to individual variation in factors such as motivation [1]. Classical conditioning has been successfully used to understand normal human learning and memory, as well as to define mechanisms associated with impaired and residual memory function associated with aging, illness or disease (see [2]). 

Veterans and active duty service members of OEF/OIF have been exposed to unprecedented risk factors for the co-morbid occurrence of PTSD and mTBI—the so-called signature injuries of these wars. Both of these “invisible wounds” [3] leaves service members vulnerable to what can become considerable associated cognitive and behavioral problems [4,5], notably memory and attention dysfunction [5,6], depression [7,8], substance abuse [9,10], anxiety and suicide [11,12]. The risk factor most commonly connected with both of these two signature injuries is exposure(s) to blast munitions. Indeed, blasts and explosions are the most common cause of injury for U.S. military personnel engaged in OEF/OIF [13], accounting for approximately 78 percent of casualties at a U.S. Army echelon II medical facility [14]. Importantly, blast-induced mTBI is *simultaneously* associated with both physical and psychological trauma [15]. The potential ramifications of co-occurring mTBI and PTSD with regard to cognitive health could be substantial; both in terms of the incidence of veterans who are suffering from deployment-related cognitive dysfunction as well as the severity of problems. Approximately 2.5 million U.S. military members have deployed to OEF/OIF and recent estimates suggest that veterans suffer rates of mTBI of 12–23 percent [16,17]. Current prevalence estimates of PTSD for OEF/OIF veterans have ranged between 11 and 18 percent [3,16,18,19], but one model projects that this rate may actually double in the coming years [20]. While research is only beginning to address the issue, convergence of these two injuries may act to exacerbate the cognitive and behavioral problems associated with each independently. Evidence supporting this notion comes from Hoge *et al*. [21] who reported that PTSD is more common among veterans who sustain a TBI compared to other injuries, as well as from civilian studies that have shown that PTSD symptoms persist longer [22] and are more severe [23] for those with mTBI compared to those without. Similarly, Lippa *et al*. [24] recently reported that in veterans seeking care following deployment, symptoms of PTSD were associated with post-concussive symptoms. 

A number of past studies have examined the influence of PTSD and mTBI on neuropsychological function, but have failed to provide a consistent picture with regard to whether, independently or combined, they exert a lasting negative influence on cognition in OEF/OIF veterans and service members (for a discussion of this complex issue see [25]). For example, findings from the Neurocognition Deployment Health Study indicated that deployment to Iraq was associated with declines in a number of cognitive domains, including verbal/visual learning and memory, but that deployment related effects remained statistically significant after taking into account intervening head injuries and changes in stress and depression symptoms ([19], see also [26]). More recently, Vasterling and her colleagues [27] found that TBI (predominantly mild) was associated only with functional impairment, while both PTSD and depression were associated with neuropsychological and functional impairment, after adjusting for TBI. Functional impairment, in this context, is defined as more subjective impairment in day-to-day performance and health. They concluded from these findings that “milder deployment-related TBI has limited lasting neuropsychological consequences in contrast to PTSD and depression, which are associated with more enduring cognitive compromise” (page 6). In a cross-sectional study, Brenner *et al*. [28] found that neither mTBI nor PTSD affected test performance in participants who were returning from their second deployment to Iraq. In contrast, Cooper *et al*. [29] reported that the presence of an mTBI did have a small but significant negative effect on performance on the Repeatable Battery for the Assessment of Neuropsychological Status (RBANS) (specifically, visuospatial construction and attention; trend for delayed memory), but psychiatric diagnosis did not influence cognitive performance. Likewise, Levin and colleagues found that verbal memory was less efficient in veterans and service members who reported a mild to moderate TBI while serving in OEF or OIF but that performance was not associated with PTSD severity [30]. In contrast, Nelson *et al*. reported that OEF/OIF veterans with comorbid mTBI and PTSD scored significantly poorer than veterans with only mTBI on measures of processing speed and executive functioning [31], suggesting that the combined effects may be particularly detrimental.

Eyeblink classical conditioning (EBCC) is one form of classical conditioning that is sensitive to the integrity of a collection of neural structures/systems that underlie a well-defined neurobiological circuit supporting the formation and expression of associative memories [32,33]. Because this circuitry is so well defined, specific patterns of performance on EBCC tasks can be linked to the integrity of the underlying component structures that make up the network in a manner that is not possible with more typical complex cognitive (neuropsychological) tasks. In its most simple form, a neutral Conditioned Stimulus (CS) (such as a tone) is paired with an Unconditioned Stimulus (US) (such as a mild puff of air to the eye) that elicits an Unconditioned, eyeblink, Response (UR). In delay eyeblink conditioning, the CS and US overlap in time and co-terminate, whereas in trace eyeblink conditioning, there is a temporal separation between the tone and the airpuff (usually between 300 ms to 1,500 ms). The trace conditioning task is more complex than the delay conditioning task because of the temporal separation between the CS and US. This temporal gap requires the formation of an abstract link or memory “trace” between the two stimuli in order for learning to occur. Regardless of the task, over the course of repeated pairings of the CS and US, participants learn an association between the CS and the US and generate an eyeblink Conditioned Response (CR) that begins prior to the onset of the US. Extinction occurs when the tone CS is presented alone following the reliable acquisition of CRs, without the US, for a number of trials whereby the production of the CR eventually drops off and is eliminated. Evidence indicates that the cerebellum houses the essential plasticity for the acquisition and storage of delay CRs [34,35] and that hippocampus (or hippocampal system) (e.g., [36] and frontal regions [37,38] play an additional essential role in trace eyeblink conditioning). We, and others, have demonstrated that performance on these two tasks differs in human clinical populations with differential damage to these neural substrates (e.g., [39,40,41,42,43,44,45,46,47,48,49,50,51,52,53]). The use of EBCC to study the influence of combat-related psychological and physical trauma on learning and memory function is limited. In fact, to date, no studies have used EBCC to elucidate possible changes in function following mTBI, either acutely or as associated with recovery or post-concussive syndrome. Eyeblink conditioning has, however, been applied to the study of PTSD but, unfortunately, it is difficult to gain a clear understanding of its effects on associative learning due to the limited number of studies and the highly varied conditioning parameters used. Vythilingam and colleagues [1] demonstrated that civilian PTSD patients were able to acquire CRs in a trace conditioning task (hippocampal-dependent) at a rate similar to healthy controls. They concluded that their results support normal hippocampally-mediated neuropsychological function in PTSD, similar to other recent reports [54,55,56]. Ayers *et al*. [57] used a delay conditioning task to investigate associative learning in combat exposed service members with and without PTSD. In that study, delay conditioning was impaired among combat veterans who were undergoing unspecified outpatient treatment, independent of PTSD status, suggesting that combat was associated with learning impairments regardless of whether PTSD is present. However, because a group of community dwelling combat veterans who were not undergoing any type of outpatient treatment were unimpaired, Ayers *et al*. concluded that variables other than prior combat must have contributed to the impaired performance of the treatment seekers. In a follow-up study, Burriss and colleagues [58] used a complex differential trace eyeblink conditioning task in combat veterans with PTSD, combat veterans without PTSD, and non-combat veterans without PTSD. Overall, veterans with PTSD showed intact differential trace conditioning and extinction. An additional finding of this study was increased reactivity among veterans with PTSD (higher UR amplitude), which reportedly did not affect EBCC learning. This may reflect hyperactive arousal among PTSD patients, similar to that observed by Ayers and colleagues above [57]. Lastly, Ginsberg *et al*. [59] examined three groups of veterans on a delay conditional discrimination eyeblink conditioning task, including combat veterans with PTSD, combat veterans without PTSD, and non-combat veterans without PTSD. In conditional discrimination learning, a compound CS is used (light and tone) to signal the onset of the US. In this study, veterans with PTSD showed impaired acquisition of CRs on reinforced trials compared to veterans without PTSD. Veterans with PTSD also demonstrated decreased CR amplitude and attenuation of the UR on reinforced trials.

As is apparent by the above review, the literature on EBCC performance in PTSD is inconclusive and varied. Overall, individuals with PTSD appear to show deficits on certain EBCC learning paradigms: delay conditional discrimination [59], possibly simple delay learning [57], intact performance on other EBCC tasks [1], and trace differential learning [58]. Collectively, these data indicate that combat exposed veterans with PTSD are impaired in acquisition using a delay paradigm but normal using a trace conditioning paradigm. These findings are unexpected, given the essential involvement of the hippocampal system in trace eyeblink conditioning together with research indicating alterations in hippocampal structure and function in PTSD [60,61]. Research examining trace conditioning performance in other clinical populations with hippocampal system involvement have revealed acquisition deficits [50]; thus, one would predict, in fact, that PTSD would also lead to impairment in trace conditioning. It is also notable that none of the studies conducted to date have directly assessed the possible effect of mTBI on EBCC performance. As reviewed above, mTBI is prevalent in OEF/OIF combat veterans and highly co-morbid with PTSD. Given that a number of studies have now indicated that the cerebellum may be especially vulnerable to blast-related damage (e.g., [62]), one might predict that blast-related mTBI would impair delay eyeblink conditioning.

The current study examined the performance of OEF/OIF deployed veterans and service members during delay and trace eyeblink conditioning using a within-subjects design (e.g., [36,63]). Additionally, all participants underwent a comprehensive evaluation for PTSD and mTBI, as well as assessment for other psychiatric comorbidities including substance abuse, anxiety and depression. Our study attempted to examine performance in four groups of OEF/OIF veterans: PTSD Only, mTBI Only, Comorbid PTSD and mTBI, and Deployed Controls. We predicted that individuals with PTSD would show differential impairment in trace compared to delay conditioning, whereas individuals with Comorbid PTSD and mTBI would show impaired conditioning in both the delay and trace tasks. As reported below, the occurrence of mTBI without co-occurring PTSD in our deployed service members was rare—only about 8%. As a result, this group was not included in the data analysis but we do present their data in Table 1, Table 2, Table 3, Table 4 for comparison.

**Table 1:** Descriptive statistics for demographic and clinical characteristics. The Overall column represents the descriptive data for the three groups.

Demographic Variables		Overall	Deployed Control	PTSD ^1^ Only	Comorbid PTSD + mTBI ^2^	mTBI Only
Age *	N	81	25	25	31	7
	Mean	35.2	37.6 *	37.3 *	31.6 *	34.4
	S.E.	1.1	2	2.1	1.4	3.2
	Minimum	20	23	21	20	22
	Maximum	62	62	58	46	46
Education **^3^**	N	81	25	25	31	7
	Mean	13.4	13.8	13.4	13	15.1
	S.E.	0.2	0.3	0.3	0.3	1
	Minimum	12	12	12	12	12
	Maximum	17	17	16	17	19
WTAR/ Estimated IQ **^4,5^**	N	80	25	25	30	7
	Mean	97.6	100.1	96.5	96.4	100.6
	S.E.	1.2	2.3	2.7	1.5	3.6
	Minimum	66	75	66 **^5^**	83	91
	Maximum	123	123	121	114	119
Gender *	Male	77	22	18	30	7
	Female	11	3	7	1	0
Ethnicity/ Race	Black or African American	4	2	1	1	0
	Hispanic or Latino	9	5	1	2	1
	American Indian	1	0	1	0	0
	Asian	1	0	1	0	0
	White	72	18	21	27	6
Smoking **^6^**	Smokers	23	4	8	11	1
	Non-smokers	62	20	17	19	6

Notes:**^ 1^** Posttraumatic Stress Disorder; **^2^** mild Traumatic Brain Injury; **^3^** General Education Development (GED) certificates were obtained by three PTSD Only participants; two Comorbid; and one Deployed Control. These individuals were given a 12 for years of education. **^4^** Wechsler Test of Adult Reading; One participant did not complete the WTAR; **^5^** The participant with the Estimated IQ score of 66 had English as a second language that likely contributed to his low score on the WTAR. He performed within normal limits on other neuropsychological measures. The next minimum score was 75; **^6^** Smoking assessments include cigarettes, cigars, and pipes; * *p* < 0.05.

**Table 2:** Gender and ethnic breakdown of study sample.

Gender:	Frequency	Percent
Male	70	86.4%
Female	11	13.6%
**Ethnicity/Race **		
Black or African American	4	4.9%
Hispanic or Latino	9	9.9%
American Indian	1	1.2%
Asian	1	1.2%
White	66	81.5%

**Table 3:** Descriptive statistics for deployment.

Deployment Variables		Overall	Deployed Control	PTSD ^1^ Only	Comorbid PTSD + mTBI ^2^	mTBI Only
Number of OEF/OIF Deployments	N	81	25	25	31	7
	Mean	1.28	1.36	1.32	1.19	1.43
	Median	1.00	1.00	1.00	1.00	1.00
	S.E.	0.056	0.114	0.111	0.072	0.202
	Minimum	1	1	1	1	1
	Maximum	3	3	3	2	2
Total Duration of Deployments (months)	N	81	25	25	31	7
	Mean	13.94	15.28	14.20	12.65	16.29
	Median	12.00	12.00	12.00	12.00	14.00
	S.E.	0.852	1.618	1.579	1.285	3.708
	Minimum	3	3	5	4	3
	Maximum	38	29	34	38	28
Time Since Last Deployment (months)	N	79	25	25	31	7
	Mean	30.652	24.20	31.04	36.16	20.57
	Median	24.00	22.00	34.00	31.00	25.00
	S.E.	2.842	3.985	4.738	5.226	4.674
	Minimum	1	1	2	1	3
	Maximum	99	80	75	99	34

Notes: **^1^** Posttraumatic Stress Disorder; **^2^** Mild Traumatic BrainInjury.

**Table 4:** Descriptive statistics for physical and emotional characteristics of study sample.

Physical/Emotional Variables		Overall	Deployed Control	PTSD ^1^ Only	Comorbid PTSD + mTBI ^2^	mTBI Only
Number of Blast Exposures	N	81	25	25	31	7
	Median	2.00	1.00	2.00	5.00	4.00
	S.E.	2.936	2.631	8.322	3.081	72.360
	Minimum	0	0	0	0	1
	Maximum	180	60	180	61	511
Number of mTBI (blast or blunt)	N	81	25	25	31	7
	Median	0.0	0.0	0.0	1.00	1.00
	S.E.	0.095	0.0	0.0	0.138	0.143
	Minimum	0	0.0	0.0	1	1
	Maximum	3	0.0	0.0	3	2
CAPS **^3^** Current ***	N	81	25	25	31	7
	Mean	51.75	17.52 ***	64.92 ***	68.74 ***	28.14
	S.E.	3.185	2.106	3.956	3.457	7.551
	Minimum	2	2	33	38	6
	Maximum	114	44	102	114	63
DASS ^4^ ***	N	81	25	25	31	7
	Mean	30.14	9.28 ***	40.16 ***	41.35 ***	19.14
	S.E.	2.873	2.091	5.694	4.4861	10.10
	Minimum	0	0	8	4	0
	Maximum	122	34	122	94	78
SMAST **^5,^^†^** (Past 12 months)	N	79	25	25	29	6
	Mean	1.59	0.64 **^†^**	2.12 **^†^**	1.97 **^†^**	0.50
	S.E.	0.297	0.305	0.667	0.482	0.342
	Minimum	0	0	0	0	0
	Maximum	12	7	12	11	2
SMAST (Lifetime)	N	51	15	16	20	4
	Mean	2.73	2.60	3.75	2.00	1.75
	S.E.	0.482	0.920	1.101	0.503	0.479
	Minimum	0	0	0	0	1
	Maximum	13	13	12	8	3
LDH **^6^** Total	N	77	25	24	28	7
	Mean	9,377.62	9,269.84	10,622.60	8,406.71	6,588.13
	S.E.	1,201.22	2,052.92	2,285.66	1,981.29	1,723.93
	Minimum	0	49.00	439.00	0.00	730.00
	Maximum	51,538.00	35,622.0	39,010.00	51,538.00	14,577.00

Notes: ^**1**^ Posttraumatic Stress Disorder; **^2^** mild Traumatic Brain Injury; **^3^** Clinician Administered PTSD Scale; **^4^** Depression, Anxiety and Stress Scale; **^5^** Short Michigan Alcohol Screening Test; **^6^** Lifetime Drinking History; **^†^** *p* < 0.09; *** *p* < 0.001.

## 2. Experimental Section

### 2.1. Participants

The first 101 service members who enrolled in the VA RR&D supported TBI Center of Excellence (CoE) at VA Boston Healthcare System: The Translational Research Center for TBI and Stress Related Disorders (TRACTS) and who completed eyeblink conditioning were eligible for this study. Thirteen of those participants had not yet deployed to Operation Enduring Freedom or Operation Iraqi Freedom (OEF/OIF) and were thus excluded, providing a final sample of 88 participants. Participants enrolled in the TRACTS CoE are recruited from throughout the Boston Metropolitan area via a full-time recruitment specialist for TRACTS who attends Yellow Ribbon Events, Task Force Meetings, and other events involving Army and Air National Guard, Marine and Marine Reserves, as well as Army and Army Reserve Units. 

The following exclusionary criteria are applied to the TRACTS study sample overall: history of seizures; prior serious medical illness, such as cerebrovascular accident, myocardial infarction, diabetes, *etc*.; current active suicidal and/or homicidal ideation, intent, or plan requiring crisis intervention; current DSM-IV diagnosis of bipolar disorder, schizophrenia or other psychotic disorder (except psychosis NOS due to trauma-related hallucinations); or cognitive disorder due to general medical condition other than TBI. Through detailed clinical interviews and consensus diagnosis procedures that are described below, participants were classified into four groups: 

*PTSD Only (n = 25)*: Participants in this group meet DSM-IV criteria for current PTSD. To meet DSM-IV PTSD diagnosis criteria, participants must score at least a “1” on frequency and “2” on severity for the requisite number of Criterion B re-experiencing symptoms (1 of a possible 5), Criterion C avoidance symptoms (3 of a possible 7), and Criterion D hyperarousal symptoms (2 of a possible 5) relating to the preceding month. Additional exclusionary criteria for this group included a deployment TBI or blast exposure that resulted in a mTBI as defined below.

*mTBI Only (n = 7)*: Participants in this group met the American Congress of Rehabilitation Medicine’s operational definition of mTBI [64], which defines a patient as having a mTBI when that person has had a traumatically induced physiological disruption of brain functioning, as manifested by at least one of the following: any period of loss of consciousness (LOC) approximately 30 min or less; any loss of memory for events immediately before or after the accident (post-traumatic amnesia; PTA) not greater than 24 h; any alteration in mental status (AMS) at the time of the accident (e.g., feeling dazed, disoriented, or confused) not greater than 24 h. This definition is consistent with that of the Department of Defense/VA common definition [65]. Mild TBI were further classified as Grade I, II or III, which was roughly adapted from Bailes and Cantu [66]. Specifically, Grade I: No LOC; and/or PTA = 0–15 min; and/or AMS = 0–15 min. Grade II: LOC ≤ 5 min; and/or PTA ≤ 24 h but > 15 min; and/or AMS ≤ 24 h but > 15 min. Grade III: LOC ≥ 5 min; and/or PTA ≥ 24 h; and/or AMS ≥ 24 h. Additional exclusionary criteria for this group included a current diagnosis for PTSD.

*Comorbid PTSD/mTBI (n = 31)*: Participants in this group met the above definition for mTBI (as defined for the mTBI Only group) and the criteria for a diagnosis of current PTSD (as defined for the PTSD Only group).

*Deployed Control (n = 25)*: Participants in this group had at least one deployment to OEF/OIF and did not meet the definition for PTSD or mTBI.

### 2.2. Procedures

All participants completed a comprehensive evaluation that took approximately eight hours to complete. If necessary, participants completed the battery across two testing sessions. Participants first underwent comprehensive assessments for TBI and PTSD. A doctoral-level psychologist administered each assessment and each case was then reviewed by at least three doctoral-level psychologists to achieve a consensus diagnoses for TBI, PTSD, and other Axis I disorders. This assessment was followed by administration of neuropsychological tests including the concurrent delay/trace eyeblink classical conditioning task, as well as self-report measures. In addition, supplemental assessment of alcohol use was obtained to provide both a quantitative and qualitative evaluation of drinking behavior.

#### 2.2.1. TBI Diagnosis

The Boston Assessment of TBI-Lifetime Version (BAT-L) is used to assess potential brain injury during three lifetime periods: pre-deployment, military deployment, and post-deployment [67]. Deployment related injuries are assessed separately for blast and blunt mechanisms of injury. TBI criteria are evaluated through open-ended questioning (to document alteration of mental status, post-traumatic amnesia, and loss of consciousness) and injuries are then graded (mild stage I, II, III; moderate; severe) according to a hybrid classification system. 

#### 2.2.2. PTSD Assessment

The presence and severity of PTSD was assessed using the Clinician-Administered PTSD Scale (CAPS). The CAPS is a semi-structured clinical interview to evaluate the frequency and intensity of re-experiencing (Criteria B), avoidance (Criteria C), and hyperarousal symptoms (Criteria D). The reliability and validity of this assessment tool are well documented [68]. Current PTSD was defined as having one re-experiencing symptom criterion, three avoidance symptoms, and two hyperarousal symptoms; these symptoms were severe enough to have significant negative impact upon functioning within the past month (in accord with DSM-IV criteria). We calculated a continuous PTSD severity score by summing the frequency and intensity ratings for symptom clusters B-C (CAPS current total score: range from 0 to 150).

#### 2.2.3. Alcohol Assessment

Lifetime Drinking History (LDH) [69] was used to estimate lifetime alcohol use. This index has been frequently used to examine the pathological effects of alcohol across the lifespan by quantifying frequency, amount, and duration of alcohol consumption during the various drinking phases of each individual’s lifespan. It also aggregates these phases yielding lifetime consumption measures. 

Self-Administered Michigan Alcohol Screening Test (SMAST) is a self-reported measure of alcoholic behavior that has been shown to provide a consistent, quantifiable method of detecting alcoholism [69]. Seltzer and colleagues suggest that a score of 0–1 on the SMAST represents a nonalcoholic profile, a score of 2 indicates a possible alcoholic profile, and a score of 3 or higher represents an alcoholic profile. 

#### 2.2.4. Substance Use Disorders

Several modules of the Structure Clinical Interview for DSM Disorders were administered, including Module E to assess for abuse and dependence of alcohol, opioid, cannabis, cocaine, amphetamine, and other substances. Two measures were created from these data to determine (1) history of alcohol abuse or dependence and (2) history of all other substance abuse or dependence. Participants were coded as having a history of alcohol abuse/dependence if they met criteria for current abuse, lifetime abuse, or both. Similarly, participants were coded as having a history of substance use other than alcohol if they met criteria for current abuse, lifetime abuse, or both.

#### 2.2.5. Concurrent Delay/Trace Eyeblink Classical Conditioning

*Apparatus*. The eyeblink conditioning apparatus that was used is a modified version of that used for EBCC in the rabbit, and was used in our most recent studies with human amnesic patients and abstinent alcoholics (e.g., [70,71]). Eyeblink responses were measured via surface EMG electrodes (Nicolet, NY, USA) placed over the orbicularis oculi muscle of the right eye. An adjustable headband was be worn by all participants to support the airpuff delivery nozzle. Data were acquired by a custom data acquisition system developed using LabView (National Instruments, Austin, TX, USA) at 5 kHz and filtered at 2 kHz using a low pass Bessel filter. Stimulus presentation and data acquisition were controlled by custom software written in LabView. EMG was digitized at 2–5 kHz. The digitized EMG signal was rectified (absolute value of the amplitude) and integrated using a decay time constant of 10 ms. The integrated-rectified signal is well correlated with eyelid closure measured with reflectance eyelid detectors [72]. 

*Stimuli.* The stimuli consisted of two clearly distinguishable tone CS’s, one 5000 Hz and one 1000 Hz, that was assigned to either the delay conditioning trials or the trace conditioning trials within subject. Assignment of the two tones was counterbalanced to the delay or trace conditioning trials across subjects. The tones were presented binaurally through stereo headphones at 85 decibels. The US was a 100 ms corneal airpuff delivered to the right eye that averaged 3 pounds of pressure per square inch. 

*Assessment of Hearing.* All participants underwent a brief audiology screening using a model 119 Beltone portable audiometer to rule out hearing loss at 300 Hz, 1000 Hz, and 5000 Hz. Each ear was tested individually. As in Solomon [73], for subjects whose threshold in either ear was between 5 and 15 dB above normal, we raised the amplitude of the CS accordingly. Subjects with greater than 15 dB hearing loss at any of these frequencies would have been replaced, but this was not necessary.

**Figure 1:**
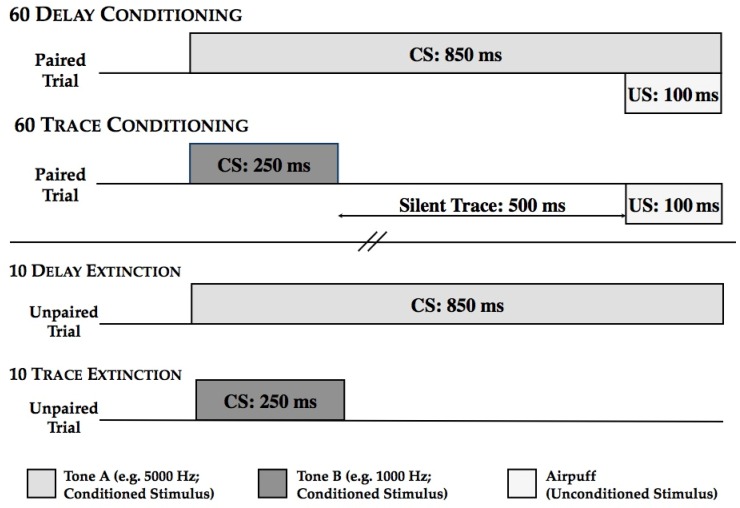
Schematic of the concurrent delay and trace task.

*EBCC Testing Procedure*. Each participant was presented with 45 delay conditioning trials and 45 trace conditioning trials randomly intermixed with the restriction that no more than three trials of either type were presented in succession. As depicted in Figure 1, delay conditioning trials consisted of an 850 ms CS followed by a 100 ms US airpuff, and both the CS and US coterminated; trace conditioning trials consisted of a 250 ms CS followed by a silent trace period of 500 ms followed by a 100 ms US airpuff. Both trial types were preceded by a 750 ms baseline period (to assess spontaneous blinks) and with an intertrial interval that varied between 8 and 12 s, with an average of 10 s. Immediately and seamlessly following the 90 conditioning trials were 18 extinction trials: nine corresponding to the delay conditioning trials and 9 corresponding to the trace conditioning trials. Thus, for delay extinction, the CS tone was presented for 850 ms and was NOT followed by the US; and for trace extinction, the CS tone was presented for 250 ms and was NOT followed by the US. These two extinction trial types were randomly intermixed with the restriction that no more than three trials of either type were presented in succession. Trial data were collapsed into nine blocks of consecutive delay and trace conditioning trials and three blocks of consecutive delay and trace extinction trials.

Participants were seated in an upright chair in a sound attenuated room and the examiner fit them with the eyeblink apparatus. The experimenter as seated in the same room, out of the direct view of the participant so that s/he could answer any questions as they arose. A compilation of Pixar short films was shown throughout the testing session to provide participants with a light distraction, a procedure that others and we have followed in many similar conditioning experiments (e.g., [50,51]). The experimenter read instructions that included the following: 

“During the experiment you will experience various stimuli including tones and puffs of air. The puff will most likely make you blink. All you are asked to do is to remain seated and blink whenever you want to. You do not need to try to hold your eye open during the airpuff. We are not doing anything to trick or deceive you. We just want to measure your responses to the tones and puffs, so respond however you would like.

We have placed a computer screen in front of you. It will play a silent movie for you to watch during testing that is intended only to provide you with some entertainment to reduce boredom.

I will remain in the room with you throughout the experiment; so if you need anything or have any questions, just let me know. If at any time you would like to quit the experiment, just let me know and we will stop immediately. Do you have any questions?

Remember, just try to relax and let your natural reflexes take over.”

*Definitions.* An eyeblink was scored as a CR if its amplitude was at least four standard deviations greater than the mean baseline response amplitude and initiated within 400 ms of US onset. Eyeblinks with latencies less than 100 ms following CS onset were recorded as alpha responses and not considered CRs [74]. The UR amplitude was used to confirm that participants were adequately stimulated to permit conditioning to occur and to assure that the unconditioned reflex was intact. The primary dependent measure was the percentage of trials on which a CR occurred; secondary measures included the onset latency of the CR, the peak latency of the CR, the amplitude of the CR, the amplitude of the UR, and the number of alpha responses. CR onset latency refers to the time at which the CR amplitude first reached 4 standard deviations above baseline. Alternatively, CR peak latency represents the time at which the given CR reached its highest amplitude. The CR amplitude is measured as peak amplitude and refers to the amount of EMG muscle activity during a CR. UR amplitude is measured as peak amplitude and refers to the amount of EMG muscle activity during the UR period, and reflects the unconditioned reflex in response to the airpuff. 

This study received approval from the VA Boston Healthcare System’s Institutional Review Board and all participants provided consent for their participation by signing an approved Informed Consent Statement.

*Data Analysis.* All data analyses have been conducted for the sample size of 81, divided into three groups: Deployed Controls, PTSD Only, and Comorbid. Version 19 of the SPSS statistical package has been used for all statistical analysis [75]. 

Separate Analysis of variance tests (ANOVA) have been conducted to assess group differences for the following measures: age, education, number of OEF/OIF deployments, total duration of each deployment (months), duration of time (measured in months) since the participants’ last deployment, summary statistics for blast exposure and military mTBI (as measure by the BAT-L questionnaire), assessment of PTSD severity (CAPS current score), current symptoms of depression, anxiety symptoms, levels of stress (measured by DASS), alcohol use history (LDH), as well as participants’ alcoholic profiles during the most recent 12 month period and lifetime periods (SMAST). Additionally, Chi-square tests were performed to examine any group differences in the incidence of diagnosed alcohol or other substance abuse and/or dependence history, gender breakdown, race/ethnicity information, and distribution of tobacco smokers.

One-sample *t*-tests were used to explore within-group differences of acquisition within each of the eyeblink conditioning tasks (Delay and Trace); mean percentage of CRs was compared to a 20% criteria, that has traditionally been utilized in other studies. Group differences in CR acquisition were compared using a repeated measures ANOVA with Group (Deployed Controls, PTSD Only, Comorbid) as a between subject factor and EBCC task (delay, trace) as a within subjects factor; age was entered as a covariate. 

Additional repeated measures ANOVA tests were used in order to confirm the occurrence of learning within the conditioning sessions. Binary classification was employed for each participant; coded as 0 if no responses occurred during the first 5 trials, and 1 if a CR was produced on each of the first 5 trials of conditioning. Group differences for secondary eyeblink conditioning measures were assessed using univariate repeated measures ANOVAs; Group was entered as a between subjects factor and age was set as a covariate.

Paired sample t-tests were used to examine the extinction of learning; mean CRs for the final nine paired conditioning trials were compared to the mean CRs for the nine extinction trials for each group. Partial correlations, controlling for age, were used to examine whether physical trauma, clinical, and alcohol measures were associated with eyeblink conditioning performance.

## 3. Results and Discussion

As noted above, the data for the relatively small mTBI group is presented for comparison purposes only; all data analysis was conducted for the sample size of 81 and for three groups: Deployed Controls, PTSD Only, and Comorbid. Demographic information for the groups is provided in Table 1. 

Analysis of variance indicated a main effect of group for age, *F*(2,78) = 3.82, *p* = 0.03, *η*^2^ = 0.09. *Post hoc* comparisons using Least Squares Difference (LSD) indicated that the Comorbid group was younger than the Deployed Controls (mean difference (MD) = −6.03, *p* < 0.05) and PTSD Only groups (MD = −5.71, *p* < 0.05). ANOVA indicated that education (F(2,78) = 1.64, *p* = 0.20, *η*^2^ = 0.04) and Estimated IQ *F*(2,78) = 0.95, *p =* 0.40, *η*^2^ = 0.02) were not significantly different across groups (F’s < 1). Chi square tests did not reveal any significant group differences for tobacco smokers, X^2^(2) = 4.9, or race/ethnicity distribution, X^2^(5) = 9.35. Gender distribution was found to be significantly different between groups, X^2^(2) = 7.3; the PTSD Only group had a higher number of females than the other groups. Frequency information regarding gender, race and ethnicity in the sample is provided in Table 2. 

Table 3 presents deployment information broken out by groups. ANOVA indicated that each of the groups were roughly equivalent with regard to the number of OEF/OIF deployments (F(2,78) = 0.84, *p =* 0.44, *η*^2^ = 0.02), total duration of each deployment (months) (F(2,78) = 0.84, *p =* 0.44, *η*^2^ = 0.02) and the total months since last deployment (F(2,78) = 1.60, *p =* 0.21, *η*^2^ = 0.04). 

Table 4 presents a subset of the data obtained from the BAT-L, including summary statistics for blast exposure and military mTBI (including blast-related and blunt force). This table additionally includes the clinical measures used to assess PTSD severity (CAPS current score), current symptoms of depression, anxiety and stress (DASS), and alcohol use history, including a characterization of participants’ alcoholic profile (SMAST) estimated for both the prior 12 months and for lifetime, and total lifetime alcohol consumption (LDH). With regard to clinical measures, only the CAPS current score (F(2,78) = 71.68, *p* = 0.001, *η*^2^ = 0.65) and the DASS (F(2,78) = 16.53, *p =* 0.001, *η*^2^ = 0.30) differed significantly across groups. For both measures, the Deployed Control group scored significantly lower than the two patient groups (CAPS: *vs*. PTSD Only, MD = −47.40, *p* < 0.001; *vs*. Comorbid, MD = −51.22, *p* < 0.001) (DASS: *vs*. PTSD Only, MD = −15.36, *p* < 0.001; *vs*. Comorbid, (MD = −16.05, *p* < 0.001). The two patient groups did not differ between each other on either CAPS current score or DASS (*p*s > 0.4). Additionally, alcohol use did not differ significantly across groups, although there was a trend in the SMAST score for the prior 12 months, *F*(2,76) = 2.50, *p =* 0.09. As reflected in Table 4, this trend was due to a greater alcoholic profile (SMAST) and greater lifetime alcohol consumption in the two patients groups compared to controls (Deployed Control *vs*. PTSD only, MD = −1.48, *p* < 0.05; Deployed Control *vs*. Comorbid, MD = −1.33, *p* < 0.07).

Chi-square analyses were performed to examine possible group differences in the incidence of diagnosed alcohol or other substance abuse and/or dependence history. For history of alcohol abuse or dependence 47 of 81 participants received a positive diagnosis but the incidence did not differ significantly across groups, X^2^(2) = 3.26. For history of substance abuse or dependence other than alcohol there were 24 of 81 participants who received a positive diagnosis but, again, the incidence did not differ significantly across groups, X^2^(2) = 2.41. 

### 3.1. Acquisition of Concurrent Delay/Trace Eyeblink Classical Conditioning

To determine whether there was significant acquisition in each task, we compared the mean percent CRs for each group separately to a 20% criteria that we used in other studies [76]. One-sample *t*-tests revealed that acquisition was significant for each task in all groups, *p* < 0.001 (Deployed Control: Delay *t*(24) = 12.86; Trace *t*(24) = 11.97. PTSD Only: Delay *t*(24) = 8.31; Trace *t*(24) = 7.56. Comorbid: Delay *t*(24) = 9.95; Trace *t*(24) = 8.71).

**Figure 2:**
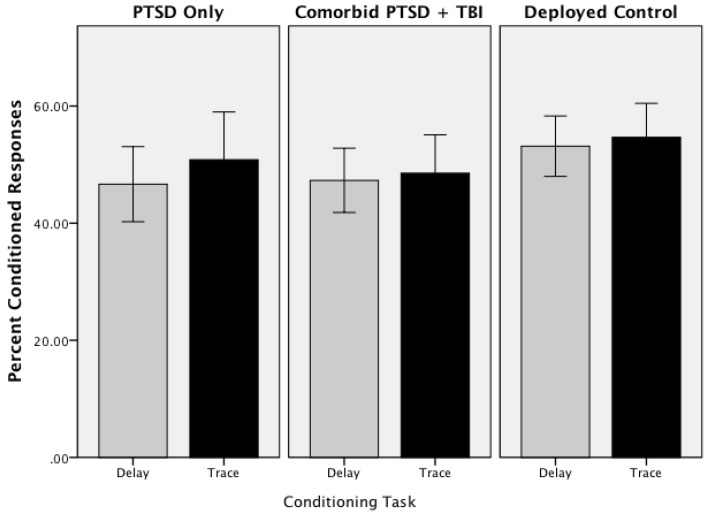
Mean percent conditioned responses for delay and trace eyeblink conditioning (error bars: +/−2 SE).

Notes: Mean percent conditioned responses for the three groups. PTSD Only, PTSD Comorbid with mTBI, and Deployed Controls. Light gray bars represent delay conditioning acquisiton, dark bar represent trace conditioning acquisition.

**Figure 3:**
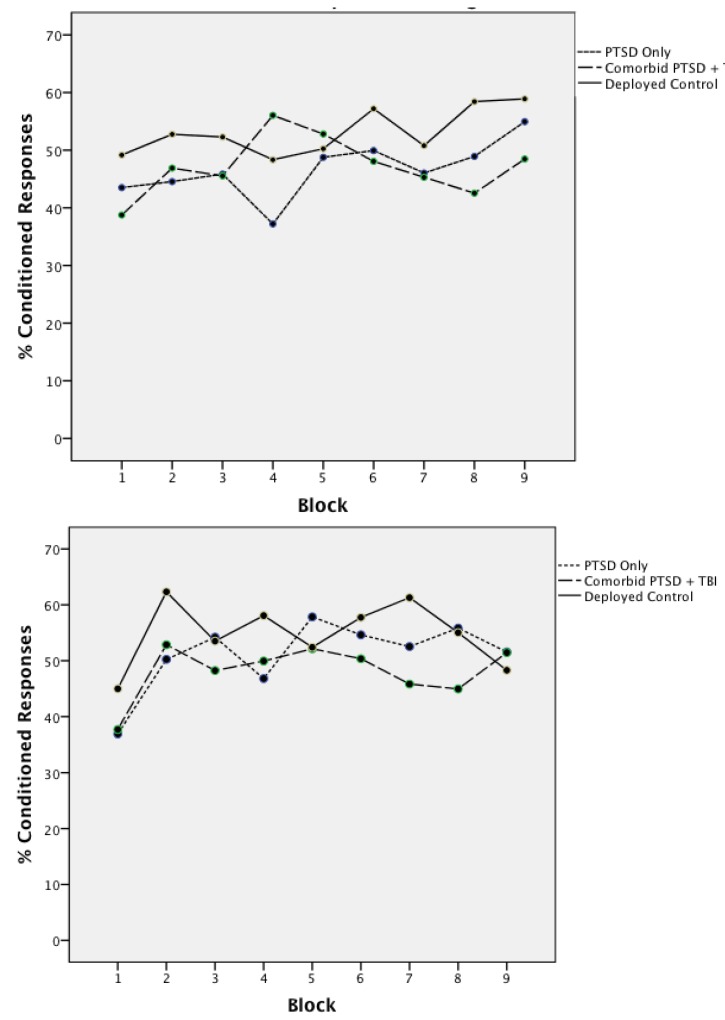
Mean percent conditioned responses by block delay conditioning.

Note: Mean percentage of conditioned responses for each group broken out by blocks of five trials each.

Group differences in acquisition of CRs was explored using a repeated measures ANOVA with Group (Deployed Controls, PTSD Only, Comorbid) as a between subject factor and EBCC task (delay, trace) as a within subjects factor; age was entered as a covariate. Mean percentages of CRs for Delay and Trace conditioning for the three groups are shown in Figure 2. ANOVA indicated that the groups acquired CRs at comparable rates across both the trace and delay task. Neither the main effect of Group (F(2,77) = 1.15, *p =* 0.32, *η*^2^ = 0.03), Task (F(1,77) = 1.18, *p =* 0.28, *η*^2^ = 0.02), nor the interaction (F(2,77) = 0.66, *p =* 0.52, *η*^2^ = 0.02) were significant. Overall, the mean percentage of CRs acquired for delay conditioning was 49.04 (SE = 1.67) and the mean percentage of CRs acquired for trace conditioning was 51.37 (SE = 2.01). Figure 3 shows the learning curves across blocks for Groups. Repeated measure ANOVA with Group, Task, and Block (1–9) (covarying for age) confirmed that the learning rate was similar across Groups with no significant main effects (Group (F(2,76) = 1.18, *p =* 0.31, *η*^2^ = 0.03), Task (F(1,76) = 1.16, *p =* 0.23, *η*^2^ = 0.02), Block (F(8,608) = 1.08, *p =* 0.38, *η*^2^ = 0.01) or interactions (Task X Block (F(8,608) = 1.42, *p* = 0.19, *η*^2^ = 0.02); Task X Block X Group (F(16,608) = 0.75, *p =* 0.75, *η*^2^ = 0.10))). Mean percent CRs for the mTBI Only group for Delay and Trace conditioning was comparable to the other groups (Delay mean = 50.48; SE = 7.25; Trace mean = 53.97; SE = 9.79). This suggests that, on average, the learning curve was relatively flat across trial blocks. To confirm that learning did occur, a second repeated measures ANOVA was conducted for the first block of trials. Each participant was coded as 0 (no) or 1 (yes) as having produced a CR on each of the first five trials. This analysis revealed a significant main effect of Trials for both Delay (F(3,312) = 2.85, *p =* 0.02, *η*^2^ = 0.04) and Trace (F(4,312) = 4.09, *p =* 0.003, *η*^2^ = 0.05) conditioning. 

A series of univariate repeated measures ANOVA’s were conducted to evaluate possible group differences in the secondary eyeblink conditioning measures (Table 5). For each dependent measure, Group (Deployed Controls, PTSD Only, Comorbid PTSD + TBI) was entered as a between subjects factor and age was entered as a covariate. Age was not found to be a significant contributor to any model and the main effect of Group was not a significant factor for any measure (Age: all *F* < 2.19; Group all *F*’s < 0.82).

**Table 5:** Summary statistics for secondary eyeblink conditioning measures (n = 81). No group differences were observed.

	Mean	Standard Error	F(2,80)
Delay CR **^1^** Onset	340.15	9.044	0.09
Delay CR Peak Latency	462.77	9.074	0.22
Delay CR Amplitude	26.05	1.72	0.73
Delay UR **^2^** Amplitude	43.36	1.89	0.08
Delay Alpha Responses	6.30	0.61	0.23
Trace CR Onset	344.03	9.80	0.35
Trace CR Peak latency	481.52	10.16	0.20
Trace CR Amplitude	29.24	1.81	0.82
Trace UR Amplitude	42.39	1.80	0.10
Trace Alpha Responses	6.21	0.55	0.58

Notes: ^**1**^ Conditioned Response; **^2^** Unconditioned Response.

### 3.2. Extinction of Concurrent Delay/Trace Eyeblink Classical Conditioning

**Figure 4:**
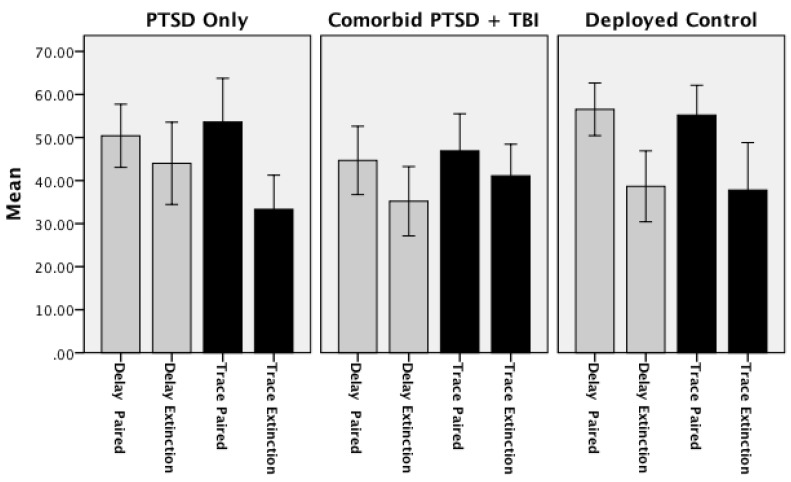
Delay and trace extinction: mean percent conditioned responses for final 3 blocks of paired trials and 3 block of extinction trials (error bars: +/−2 SE).

Notes: Extinction mean percentage of conditioned responses for each group for the final three acquisition blocks and the three extinction blocks. Light gray bars represent delay conditioning acquisiton, dark bar represent trace conditioning acquisition.

Extinction of learning was evaluated by paired t-tests comparing the mean CRs for the final nine paired conditioning trials to the mean CRs for the nine extinction trials within each group separately. The Deployed Control group demonstrated significant extinction of Delay CRs (*t*(24) = 3.86; *p =* 0.001); and fewer CRs during extinction trials for paired Trace trials as compared to extinction trials, although the difference was not significant (*t*(24) = 21.30; *p =* 0.205). The lack of significant extinction for Trace conditioning appeared to be due to a drop in conditioning performance across the final nine conditioning trials for this group as opposed to a high rate of CRs produced during extinction. The PTSD Only group produced significantly fewer CRs in the Trace conditioning task (*t*(24) = 2.52; *p* < 0.02) and a trend for extinction in the Delay conditioning task (*t*(24) = 1.93, *p* = 0.07). Lastly, the Comorbid group produced significantly fewer CRs in the Delay conditioning task (*t*(29) = 2.10; *p* = 0.04), but not in the Trace conditioning task (*t*(24) = 0.07; *p* = 0.94). These data would suggest a pattern of extinction in both the Deployed Control and PTSD Only in both delay and trace conditioning, extinction for delay CRs for the Comorbid group in Delay conditioning but a clear impairment for the Comorbid group to extinguish Trace CRs (see Figure 4 and Table 6).

**Table 6:** Mean (and Standard Error) percent Conditioned responses for the final nine paired conditioning trials and nine extinction (tone alone) trials for delay and trace conditioning.

	Delay Conditioning		Trace Conditioning	
	Final 9 Paired Trials	Extinction Trials	Final 9 Paired Trials	Extinction Trials
Deployed Controls ***	58.22	38.67 ***	46.22	37.78
	(3.86)	(4.11)	(4.04)	(5.52)
PTSD Only ^†,^*	53.33	44.00 **^†^**	49.33	33.33 *
	(3.14)	(4.78)	(5.63)	(3.95)
Comorbid PTSD/TBI *	45.16 *	35.48	41.21	40.86
	(4.15)	(3.90)	(4.87)	(3.55)

Notes: **^†^*** p* < 0.09; * *p* < 0.05; *** *p* < 0.001.

### 3.3. Associations between Conditioning Performance with Physical and Emotional Measures of Trauma and Deployment Statistics

**Figure 5:**
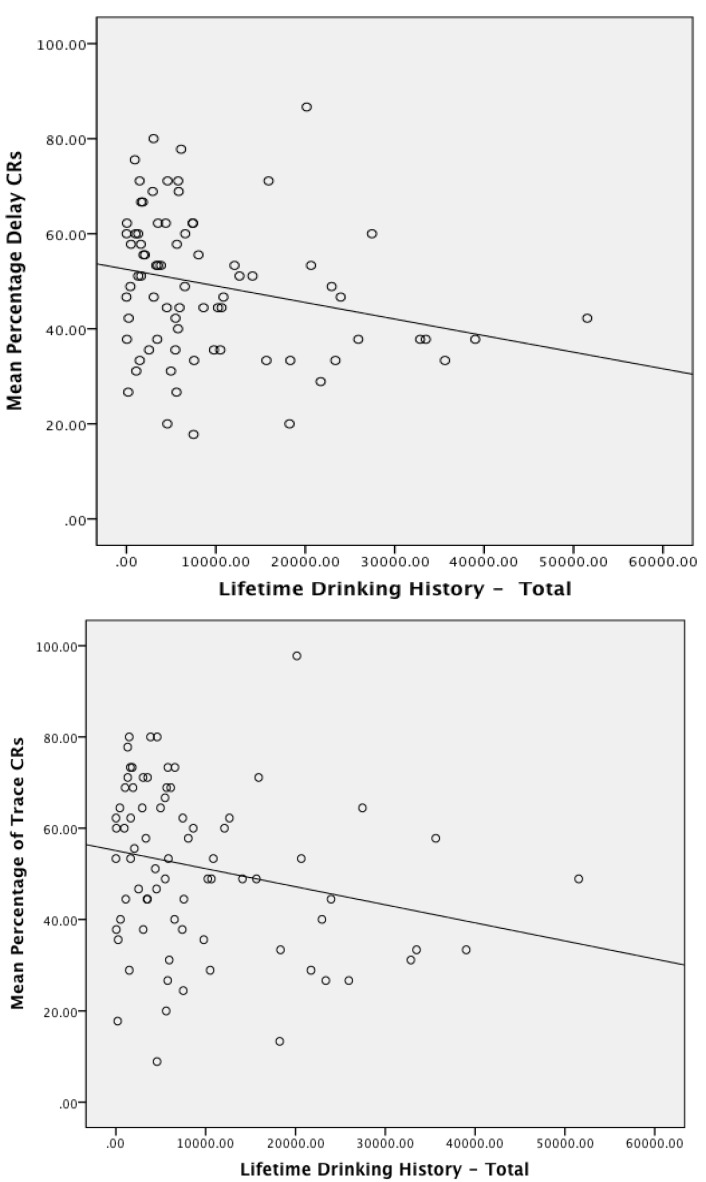
Scatterplots of the mean percentage of conditoned reponses for Delay (**a**) and Trace (**b**) as a function of lifetime history of alcohol use (total quantitiy consumed).

In a final analysis, partial correlations, controlling for age, were used to determine whether measures of physical trauma (blast exposures, mTBI) and clinical measures (CAPS score), depression, stress and anxiety (DASS score), and alcohol use (LDH and SMAST) were predictive of eyeblink conditioning performance. Only measures of alcohol use significantly predicted eyeblink conditioning performance. Specifically, Delay conditioning (mean percent CRs) was associated with SMAST-Lifetime (*r*(48) = −0.390, *p* = 0.005), LDH Total (*r*(74) = −0.271, *p* = 0.018), and to a lesser extent, SMAST for the prior 12 month period immediately preceding the test date (*r*(76) = −0.205, *p* = 0.072). Trace conditioning was associated with SMAST-Lifetime (*r*(48) = −0.440, *p* = 0.001), SMAST score for the prior 12 month period (*r*(76) = −0.231, *p* = 0.041), and LDH Total (*r*(74) = −0.242, *p* = 0.035). Figure 5 displays the relationship between LDH-Total and mean percent CRs for delay conditioning 5a and trace conditioning 5b. These figures clearly show the negative association between quantities of alcohol consumed throughout the lifetime and eyeblink conditioning performance. Indeed, an ANOVA using the median split on LDH total (median LDH-Total = 5,643.00) as a between subjects factor (covarying for age) revealed a significant difference in trace conditioning performance between the upper and lower split, *F*(1, 74) = 4.33, *p* < 0.05). The mean percentage of trace CRs for the lower half was 55.27 (SE = 2.90) and for the upper half was 47.19 (SE = 2.94). Delay conditioning performance did not differ in this analysis, *F*(1,74) = 1.83. The mean percentage of delay CRs for the lower half was 51.28 (SE = 2.45) and for the upper half was 47.13 (SE = 2.49).

Three primary findings emerged from this study examining concurrent delay and trace eyeblink conditioning in military veterans. First, deployed veterans from OEF/OIF who have a clinical diagnosis of PTSD or who have a diagnosis of PTSD with comorbid mTBI acquired delay and trace classically conditioned responses to levels and rates similar to deployed veterans who do not have these diagnoses. This documents the ability of our participants to acquire basic associative responses regardless of diagnosis. Second, we found evidence to suggest a differential impairment of extinction in the two clinical groups: the PTSD Only group had impaired extinction for delay CRs but not trace CRs, whereas the Comorbid PTSD and mTBI group had impaired extinction for trace CRs but not delay CRs. The deployed control group evidenced successful extinction for both delay and trace CRs. Third, acquisition of CRs for both delay and trace conditioning, as well as extinction of trace CRs was negatively associated with alcoholic behavior across all participants. 

### 3.4. Acquisition of Delay and Trace Conditioned Responses

All three groups, deployed controls, PTSD only, and Comorbid PTSD with mTBI, were able to acquire delay and trace conditioning in parallel to similar levels and at similar rates. The overall mean percentage of CRs observed, approximately 49% for delay and 51% for trace, is actually superior to that observed in prior studies using a concurrent delay and trace acquisition task [36,63]. These data suggest that the neural systems necessary for the acquisition of delay and trace eyeblink conditioned responses are sufficiently intact to support successful acquisition in the face of PTSD and comorbid PTSD with mTBI. Specifically, these data suggest that the cerebellar circuitry necessary for the acquisition of all eyeblink conditioned responses is sufficiently preserved to support these basic associative learning tasks, and that the forebrain system necessary for acquisition of trace conditioned responses is similarly preserved. These findings are consistent with some reports in the literature using a trace conditioning paradigm [1], trace differential learning [58], and suggest that delay conditioning maybe also be intact in PTSD, at least given the parameters of the concurrent delay/trace task used in the present study.

Other metrics of conditioning performance, including CR onset latency, CR peak latency and amplitude, and UR amplitude indicated that the two patient groups were similar to the deployed control group with regard to the timing and magnitude of both conditioned and unconditioned responses. We did not see evidence of increased CR or UR amplitude in this study, possibly indicative of increased reactivity associated with PTSD, as has been reported [58]. It is concluded from these data that a diagnosis of PTSD, either alone or with comorbid mTBI does not negatively influence the acquisition of simple associative responses as assessed with simple delay and trace eyeblink classical conditioning. Correlational analyses support this conclusion, as neither PTSD severity (CAPS score) nor the number of mTBI’s were significantly related to acquisition. These data add to, and may help to clarify, an existing literature that to this point has not provided a consistent picture of the integrity of behavioral and neural systems associated with eyeblink classical conditioning. 

### 3.5. Extinction of Delay and Trace Conditioned Responses

In contrast to being able to acquire CRs normally, patient groups showed a differential pattern of deficits in the extinction of CRs. Specifically, the PTSD only group did not show a significant reduction in CRs during extinction compared to the final blocks of paired trials for delay CRs but did show significant extinction for trace CRs, whereas the comorbid PTSD group showed the opposite pattern: significant extinction for delay conditioned responses but not for trace conditioned responses. These findings are interesting from a methodological standpoint because they indicate that extinction can be specific to a particular CS (*i.e.*, extinction discrimination), even under such challenging conditions as was present in this study that is parallel acquisition and extinction. Recall, that during the extinction phase, as during the acquisition phase, delay and trace CSs (two different tones that were counterbalance to delay and trace conditions across subjects) were randomly intermixed. 

The literature regarding the status of extinction of eyeblink CRs in PTSD is very limited, especially with regard to the extinction of delay CRs. Ayers *et al*. [57] did include extinction trials in their delay conditioning study, but learning was so poor during the acquisition phase the extinction data presented are not conclusive. Unfortunately, the complex discrimination delay study by Ginsberg and colleagues did not assess extinction [59]. However, both Vythilingam *et al*. [1] and Burriss *et al*. [58] reported intact extinction in their trace conditioning tasks, which is consistent with the trace extinction data for our PTSD only group. 

As this was the first use of the classical conditioning paradigm to examine associative learning using EBCC to differentiate patients with PTSD alone from patients with both PTSD and mTBI, the apparent double dissociation observed in the extinction data requires replication and data clarifying its implication for the neuropathological basis of these conditions. However, a recent study by Kalmbach and Mauk [77] may provide a theoretical context in which such a dissociation could occur. It has been previously established that extinction of delay eyeblink responses depends on the integrity of the same neural structures in the cerebellum that are responsible for acquisition; that is, the deep cerebellar nuclei and cortex [78]. Kalmback and Mauk provided evidence to suggest that extinction of trace CRs may similarly be mediated by the two primary sites that support trace acquisition, one in the cerebellum and one in forebrain structures. A possible explanation of our data then is that the delay conditioning extinction deficit observed in the PTSD only group may have been due to impairment in cerebellum function (possibly related to multiple blast exposures [62]) while successful trace extinction may have been supported by intact forebrain systems that could compensate for the cerebellum impairment. In contrast, the impaired trace extinction but intact delay extinction in the Comorbid PTSD and mTBI group may have been due to compromised forebrain function (impairing trace) but intact cerebellum function (preserving delay). A problem with this explanation is that it is unclear why the Comorbid group would not have shown an extinction deficit in delay as well, as they had similar levels of blast exposure as the PTSD only group. Nevertheless, the model proposed does provide a framework with which to begin to understand this complex phenomenon. 

Lastly, the extinction findings reported here suggest that the commonly observed failure to extinguish learned responses previously observed in PTSD are not limited to fear memories and may, in fact, extend to failure to extinguish simple motor responses. Pavlovian fear conditioning has been a very important model system in understanding the neurobiological mechanisms of PTSD. Indeed, PTSD is often conceptualized as a disease of learning and memory in which fear responses are amplified and fail to extinguish. The fact that extinction deficits were observed in both of the patient groups in this study suggests that the extinction failure in PTSD may not be limited to fear memories and implicates a more generalized extinction deficit possibly attributable to dysfunction in medial prefrontal cortex [37,79,80,81]. 

### 3.6. Association of Alcohol Use and Performance on Eyeblink Classical Conditioning

Alcohol use, both as a quantitative measure and a qualitative measure, was negatively associated with the acquisition of delay and trace eyeblink conditioning. These findings extend a number of previous studies in our laboratory documenting alcohol-related eyeblink conditioning impairments in *abstinent chronic alcoholics* (e.g., [51,52,76]) to a relatively young, non-abstinent sample. Not only did we observe the negative association, we also found a significant impairment in trace conditioning between relatively heavy lifetime drinkers versus light lifetime drinkers (as determined by a median split of the LDH Total measure). As we have suggested in the past, these associations and impairments likely reflect alcohol-related degeneration of the cerebellum and forebrain structures that support associative learning. For example, a large literature exists documenting the negative effect of alcohol on the brain, including neuronal degeneration of the cortex, cerebellum, brainstem, and changes in the integrity of cerebral white matter (e.g., [82,83,84,85,86,87]). More recent data has linked alcohol use with widespread reductions in cortical thickness in a group of community dwelling former alcoholics [88]. 

## 4. Conclusions

These data demonstrate intact acquisition of delay and trace eyeblink conditioned responses in service members and veterans with PTSD only and Comorbid PTSD with mTBI. However, deficits in the extinction of conditioned responses were observed that might suggest a generalized impairment in PTSD in the ability to extinguish associative memories. Conditioning performance (both with regard to acquisition and extinction) was moderated by alcohol use and may be indicative of early structural brain changes in this cohort. The fact that alcohol use was associated with cognitive impairment in this relatively young cohort is alarming and has important implications for individuals with PTSD and/or mTBI. Alcohol is a common method of self-medication for individuals with PTSD and/or mTBI, but the consequences of its use, particularly over the lifetime of these young individuals, could be devastating.

## References

[1] Vythilingam M., Lawley M., Collin C., Bonne O., Agarwal R., Hadd K., Charney D.S., Grillon C. (2006). Hydrocortisone impairs hippocampal-dependent trace eyeblink conditioning in post-traumatic stress disorder. Neuropsychopharmacology.

[2] Woodruff-Pak D.S., Steinmetz J.E. (2000). Eyeblink Classical Conditioning: Volume I—Applications in Humans.

[3] Tanielian T., Jaycox L.H. (2008). Invisible Wounds of War: Psychological and Cognitive Injuries, Their Consequences, and Services to Assist Recovery.

[4] Stein M.B., McAllister T.W. (2009). Exploring the convergence of posttraumatic stress disorder and mild traumatic brain injury. Amer. J. Psychiat..

[5] Vasterling J.J., Verfaellie M., Sullivan K.D. (2009). Mild traumatic brain injury and posttraumatic stress disorder in returning veterans: Perspectives from cognitive neuroscience. Clin. Psychol. Rev..

[6] Vanderploeg R.D., Belanger H.G., Curtiss G. (2009). Mild traumatic brain injury and posttraumatic stress disorder and their associations with health symptoms. Arch. Phys. Med. Rehabil..

[7] Kim E., Lauterbach E.C., Reeve A., Arciniegas D.B., Coburn K.L., Mendez M.F., Rummans T.A., Coffey E.C. (2007). Neuropsychiatric complications of traumatic brain injury: A critical review of the literature (a report by the ANPA committee on research). J. NNeuropsychiatr. Clin. Neurosc..

[8] National R.C. (2008). Long-term Consequences of Traumatic Brain Injury. Gulf War and Health.

[9] Graham D.P., Cardon A.L. (2008). An update on substance use and treatment following traumatic brain injury. Ann. N. Y. Acad. Sci..

[10] Mills K.L., Teesson M., Ross J., Peters L. (2006). Trauma, ptsd, and substance use disorders: Findings from the australian national survey of mental health and well-being. Amer. J. Psychiat..

[11] Desai R.A., Dausey D., Rosenheck R.A. (2008). Suicide among discharged psychiatric inpatients in the department of veterans affairs. Mil. Med..

[12] Gutierrez P.M., Brenner L.A., Huggins J.A. (2008). A preliminary investigation of suicidality in psychiatrically hospitalized veterans with traumatic brain injury. Arch. Suicide Res..

[13] Warden D. (2006). Military tbi during the iraq and afghanistan wars. J. Head Trauma Rehabil..

[14] Murray C.K., Reynolds J.C., Schroeder J.M., Harrison M.B., Evans O.M., Hospenthal D.R. (2005). Spectrum of care provided at an echelon ii medical unit during operation iraqi freedom. Mil. Med..

[15] Morrow C.E., Bryan C.J., Isler W.C. (2011). Concussive and psychological symptom predictors of aeromedical evacuation following possible brain injury among deployed military personnel. Psychol. Serv..

[16] Schneiderman A.I., Braver E.R., Kang H.K. (2008). Understanding sequelae of injury mechanisms and mild traumatic brain injury incurred during the conflicts in iraq and afghanistan: Persistent postconcussive symptoms and posttraumatic stress disorder. Amer. J. Epidemiol..

[17] Terrio H., Brenner L.A., Ivins B.J., Cho J.M., Helmick K., Schwab K., Scally K., Bretthauer R., Warden D. (2009). Traumatic brain injury screening: Preliminary findings in a U.S. army brigade combat team. J. Head Trauma Rehabil..

[18] Hoge C.W., Castro C.A., Messer S.C., McGurk D., Cotting D.I., Koffman R.L. (2004). Combat duty in iraq and afghanistan, mental health problems, and barriers to care. N. Engl. J. Med..

[19] Vasterling J.J., Proctor S.P., Amoroso P., Kane R., Heeren T., White R.F. (2006). Neuropsychological outcomes of army personnel following deployment to the iraq war. JAMA.

[20] Atkinson M.P., Guetz A., Wein L.M. (2009). A dynamic model for post traumatic stress disorder among us troops in operation iraqi freedom. Manag. Sci..

[21] Hoge C.W., McGurk D., Thomas J.L., Cox A.L., Engel C.C., Castro C.A. (2008). Mild traumatic brain injury in U.S. Soldiers returning from iraq. N. Engl. J. Med..

[22] Harvey A.G., Bryant R.A. (2000). Two-year prospective evaluation of the relationship between acute stress disorder and posttraumatic stress disorder following mild traumatic brain injury. Amer. J. Psychiat..

[23] Chemtob C.M., Muraoka M.Y., Wu-Holt P., Fairbank J.A., Hamada R.S., Keane T.M. (1998). Head injury and combat-related posttraumatic stress disorder. J. Nerv. Ment. Dis..

[24] Lippa S.M., Pastorek N.J., Benge J.F., Thornton G.M. (2010). Postconcussive symptoms after blast and nonblast-related mild traumatic brain injuries in afghanistan and iraq war veterans. J. Int. Neuropsychol. Soc..

[25] Vasterling J.J., Dikmen S. (2012). Mild traumatic brain injury and posttraumatic stress disorder: Clinical and conceptual complexities. J. Int. Neuropsychol. Soc..

[26] Marx B.P., Brailey K., Proctor S.P., Macdonald H.Z., Graefe A.C., Amoroso P., Heeren T., Vasterling J.J. (2009). Association of time since deployment, combat intensity, and posttraumatic stress symptoms with neuropsychological outcomes following iraq war deployment. Arch. Gen. Psychiat..

[27] Vasterling J.J., Brailey K., Proctor S.P., Kane R., Heeren T., Franz M. (2012). Neuropsychological outcomes of mild traumatic brain injury, post-traumatic stress disorder and depression in iraq-deployed us army soldiers. Br. J. Psychiat..

[28] Brenner L.A., Terrio H., Homaifar B.Y., Gutierrez P.M., Staves P.J., Harwood J.E., Reeves D., Adler L.E., Ivins B.J., Helmick K. (2010). Neuropsychological test performance in soldiers with blast-related mild TBI. Neuropsychology.

[29] Cooper D.B., Mercado-Couch J.M., Critchfield E., Kennedy J., Vanderploeg R.D., DeVillibis C., Gaylord K.M. (2010). Factors influencing cognitive functioning following mild traumatic brain injury in OIF/OEF burn patients. Neurorehabilitation.

[30] Levin H.S., Wilde E., Troyanskaya M., Petersen N.J., Scheibel R., Newsome M., Radaideh M., Wu T., Yallampalli R., Chu Z. (2010). Diffusion tensor imaging of mild to moderate blast-related traumatic brain injury and its sequelae. J. Neurotrauma.

[31] Nelson L.A., Yoash-Gantz R.E., Pickett T., Campbell T.A. (2009). Relationship between processing speed and executive functioning performance among OIF/OEF veterans: Implications for postdeployment rehabilitation. J. Head Trauma Rehabil..

[32] Thompson R.F. (1986). The neurobiology of learning and memory. Science.

[33] Thompson R.F. (1988). The neural basis of basic associative learning of discrete behavioral responses. Trends Neurosci..

[34] Kim J.J., Thompson R.F. (1997). Cerebellar circuits and synaptic mechanisms involved in classical eyeblink conditioning. Trends Neurosci..

[35] Raymond J.L., Lisberger S.G., Mauk M.D. (1996). The cerebellum: A neuronal learning machine?. Science.

[36] Cheng D.T., Disterhoft J.F., Power J.M., Ellis D.A., Desmond J.E. (2008). Neural substrates underlying human delay and trace eyeblink conditioning. Proc. Natl. Acad. Sci. USA.

[37] Weible A.P., McEchron M.D., Disterhoft J.F. (2000). Cortical involvement in acquisition and extinction of trace eyeblink conditioning. Behav. Neurosci..

[38] McLaughlin J., Skaggs H., Churchwell J., Powell D.A. (2002). Medial prefrontal cortex and pavlovian conditioning: Trace versus delay conditioning. Behav. Neurosci..

[39] Arndt T.L., Stodgell C.J., Rodier P.M. (2005). The teratology of autism. Int. J. Dev. Neurosci..

[40] Bolbecker A.R., Mehta C.S., Edwards C.R., Steinmetz J.E., O’Donnell B.F., Hetrick W.P. (2009). Eye-blink conditioning deficits indicate temporal processing abnormalities in schizophrenia. Schizophr. Res..

[41] Bolbecker A.R., Steinmetz A.B., Mehta C.S., Forsyth J.K., Klaunig M.J., Lazar E.K., Steinmetz J.E., O’Donnell B.F., Hetrick W.P. (2011). Exploration of cerebellar-dependent associative learning in schizophrenia: Effects of varying and shifting interstimulus interval on eyeblink conditioning. Behav. Neurosci..

[42] Brown S.M., Kieffaber P.D., Carroll C.A., Vohs J.L., Tracy J.A., Shekhar A., O’ÄôDonnell B.F., Steinmetz J.E., Hetrick W.P. (2005). Eyeblink conditioning deficits indicate timing and cerebellar abnormalities in schizophrenia. Brain Cognition.

[43] Coffin J.M., Baroody S., Schneider K., O’Neill J. (2005). Impaired cerebellar learning in children with prenatal alcohol exposure: A comparative study of eyeblink conditioning in children with adhd and dyslexia. Cortex.

[44] Edwards C.R., Newman S., Bismark A., Skosnik P.D., O’Donnell B.F., Shekhar A., Steinmetz J.E., Hetrick W.P. (2008). Cerebellum volume and eyeblink conditioning in schizophrenia. Psychiatry Res. Neuroimaging.

[45] Fortier C., Maksimovskiy A.L., Venne J.R., LaFleche G., McGlinchey R.E. (2009). Silent trace eliminates differential eyeblink learning in abstinent alcoholics. Int. J. Environ. Res. Public Health.

[46] Fortier C.B., Disterhoft J.D., McGlinchey-Berroth R. (2000). Cerebellar cortical degeneration disrupts discrimination learning but not delay or trace eyeblink conditioning. Neuropsychology.

[47] Gabrieli J.D.E., McGlinchey-Berroth R., Carrillo M.C., Gluck M.A., Cermak L.S., Disterhoft J.F. (1995). Intact delay-eyeblink classical conditioning in amnesia. Behav. Neurosci..

[48] Jacobson S.W., Stanton M.E., Dodge N.C., Pienaar M., Fuller D.S., Molteno C.D., Meintjes E.M., Hoyme H.E., Robinson L.K., Khaole N. (2011). Impaired delay and trace eyeblink conditioning in school-age children with fetal alcohol syndrome. Alcohol. Clin. Exp. Res..

[49] Jacobson S.W., Stanton M.E., Molteno C.D., Burden M.J., Fuller D.S., Hoyme H.E., Robinson L.K., Khaole N., Jacobson J.L. (2008). Impaired eyeblink conditioning in children with fetal alcohol syndrome. Alcohol.: Clin. Exp. Res..

[50] McGlinchey-Berroth R., Carrillo M.C., Gabrieli J.D.E., Brawn C.M., Disterhoft J.F. (1997). Impaired trace eyeblink conditioning in bilateral medial temporal lobe amnesia. Behav. Neurosci..

[51] McGlinchey-Berroth R., Cermak L.S., Carrillo M., Armfield S., Gabrieli J.D.E., Disterhoft J.F. (1995). Impaired delay eyeblink conditioning in amnesic korsakoff’s patients and recovered alcoholics. Alcohol.: Clin. Exp. Res..

[52] McGlinchey-Berroth R., Fortier C., Tangle L., Disterhoft J.F. (2000). Trace eyeblink conditioning in naïve and trained recovered alcoholics. Soc. Neurosci. Abstr..

[53] Oristaglio J., Hyman West S., Ghaffari M., Lech M.S., Verma B.R., Harvey J.A., Welsh J.P., Malone R.P. (2013). Children with autism spectrum disorders show abnormal conditioned response timing on delay, but not trace, eyeblink conditioning. Neuroscience.

[54] Neylan T.C., Lenoci M., Rothlind J., Metzler T.J., Schuff N., Du A.T., Franklin K.W., Weiss D.S., Weiner M.W., Marmar C.R. (2004). Attention, learning, and memory in posttraumatic stress disorder. J. Trauma. Stress.

[55] Pederson C.L., Maurer S.H., Kaminski P.L., Zander K.A., Peters C.M., Stokes-Crowe L.A., Osborn R.E. (2004). Hippocampal volume and memory performance in a community-based sample of women with posttraumatic stress disorder secondary to child abuse. J. Trauma. Stress.

[56] Vasterling J.J., Brailey K., Constans J.I., Sutker P.B. (1998). Attention and memory dysfunction in posttraumatic stress disorder. Neuropsychology.

[57] Ayers E.D., White J., Powell D.A. (2003). Pavlovian eyeblink conditioning in combat veterans with and without post-traumatic stress disorder. Integr. Physiol. Behav. Sci..

[58] Burriss L., Ayers E., Powell D.A. (2007). Combat veterans show normal discrimination during differential trace eyeblink conditioning, but increased responsivity to the conditioned and unconditioned stimulus. J. Psychiatr. Res..

[59] Ginsberg J.P., Ayers E., Burriss L., Powell D.A. (2008). Discriminative delay pavlovian eyeblink conditioning in veterans with and without posttraumatic stress disorder. J. Anxiety Disord..

[60] Bremner J.D., Elzinga B., Schmahl C., Vermetten E. (2008). Structural and functional plasticity of the human brain in posttraumatic stress disorder. Prog. Brain Res..

[61] Gilbertson M.W., Shenton M.E., Ciszewski A., Kasai K., Lasko N.B., Orr S.P., Pitman R.K. (2002). Smaller hippocampal volume predicts pathologic vulnerability to psychological trauma. Nat. Neurosci..

[62] Donald C.L.M., Johnson A.M., Cooper D., Nelson E.C., Werner N.J., Shimony J.S., Snyder A.Z., Raichle M.E., Witherow J.R., Fang R. (2011). Detection of blast-related traumatic brain injury in U.S. Military personnel. N. Engl. J. Med..

[63] Cheng D.T., Faulkner M.L., Disterhoft J.F., Desmond J.E. (2010). The effects of aging in delay and trace human eyeblink conditioning. Psychol. Aging.

[64] Committee M.T.B.I. (1993). Definition of mild traumatic brain injury. J. Head Trauma Rehabil..

[65] Department of Veterans Affairs Office of Quality and Performance and Department of Defense Quality Management Directorate, USAMC (2009). VA/DOD Clinical Practice Guidelines for Management Of Concussion/Mild Traumtic Brain Injury.

[66] Bailes J.E., Cantu R.C. (2001). Head injury in athletes. Neurosurgery.

[67] Fortier C.B., Amick M.M., Grande L., McGlynn S., Kenna A., Morra L., Clark A., Milberg W.P., McGlinchey R.E. (2013). The boston assessment of traumatic brain injury-lifetime (BAT-L) semistructured interview: Evidence of research utility and validity. J. Head Trauma Rehabil..

[68] Blake D.D., Weathers F.W., Nagy L.M., Kaloupek D.G., Gusman F.D., Charney D.S., Keane T.M. (1995). The development of a clinician-administered ptsd scale. J. Trauma. Stress.

[69] Skinner H.A., Sheu W.J. (1982). Reliability of alcohol use indices: The lifetime drinking history and the mast. J. Stud. Alcohol.

[70] Fortier C.B., Steffen E.M., LaFleche G., Venne J.R., Disterhoft J.F., McGlinchey R.E. (2008). Delay discrimination and reversal eyebink classical conditioning in abstinent chronic alcoholics. Neuropsychology.

[71] McGlinchey R.E., Capozzi S., Fortier C.B., Disterhoft J.F. (2008). Procedural memory system supports single cue trace eyeblink conditioning in medial temporal lobe amnesia. Neuropsychology.

[72] Knuttinen M.G., Power J.M., Preston A.R., Disterhoft J.F. (2001). Awareness in classical differential eyeblink conditioning in young and aging humans. Behav. Neurosci..

[73] Solomon P.R., Pomerleau D., Morse D.L. (1989). Acquisition of the classically conditioned eyeblink response in humans over the life span. Psychol. Aging.

[74] Gormezano I., Sidowski J.B. (1966). Classical Conditioning. Experimental Methods and Instrumentation in Psychology.

[75] Corp I. (2010). Ibm Spss Statistics for Macintosh.

[76] McGlinchey R., Fortier C.B., Capozzi S., Disterhoft J.F. (2005). Trace eyeblink conditioning in abstinent alcoholics: Effects of complex task demands and prior conditioning. Neuropsychology.

[77] Kalmbach B.E., Mauk M.D. (2012). Multiple sites of extinction for a single learned response. J. Neurophysiol..

[78] Robleto K., Poulos A.M., Thompson R.F. (2004). Brain mechanisms of extinction of the classically conditioned eyeblink response. Learn. Memory.

[79] McCormick D.A., Thompson R.F. (1982). Locus coeruleus lesions and resistance to extinction of a classically conditioned response: Involvement of the neocortex and hippocampus. Brain Res..

[80] Morgan M.A., Romanski L.M., LeDoux J.E. (1993). Extinction of emotional learning: Contribution of medial prefrontal cortex. Neurosci. Lett..

[81] Myers K.M., Davis M. (2007). Mechanisms of fear extinction. Mol. Psychiatr..

[82] Fein G., di Sclafani V., Cardenas V.A., Goldmann H., Tolou-Shams M., Meyerhoff D.J. (2002). Cortical gray matter loss in treatment-naive alcohol dependent individuals. Alcohol. Clin. Exp. Res..

[83] Harper C. (1982). Neuropathology of brain damage caused by alcohol. Med. J. Australia.

[84] Harper C., Kril J., Daly J. (1987). Are we drinking our neurons away?. Br. Med. J..

[85] Jernigan T.L., Schafer K., Butters N., Cermak L.S. (1991). Magnetic resonance imaging of alcoholic korsakoff patients. Int. J. Neuropsychopharmacol..

[86] Pfefferbaum A., Sullivan E.V. (2002). Microstructural but not macrostructural disruption of white matter in women with chronic alcoholism. Neuroimage.

[87] Pfefferbaum A., Sullivan E.V., Hedehus M., Adalsteinsson E., Lim K.O., Moselesy M. (2000). In vivo detection and functional correlates of white matter microstructural disruption in chronic alcoholism. Alcohol. Clin. Exp. Res..

[88] Fortier C.B., Leritz E.C., Salat D.H., Venne J.R., Maksimovskiy A.L., Williams V., Milberg W.P., McGlinchey R.E. (2011). Reduced cortical thickness in abstinent alcoholics and association with alcoholic behavior. Alcohol. Clin. Exp. Res..

